# Reducing caesarean section rate in an urban hospital serving women attending privately in India – a quality improvement initiative

**DOI:** 10.1186/s12884-020-03234-x

**Published:** 2020-09-23

**Authors:** Abhishek Bhartia, Rinku Sen Gupta Dhar, Saru Bhartia

**Affiliations:** Sitaram Bhartia Institute of Science and Research, Delhi, India

**Keywords:** Maternity care, Caesarean section rate, Quality improvement

## Abstract

**Background:**

In line with global trends, India has witnessed a sharp rise in caesarean section (CS) deliveries, especially in the private sector. Despite the urgent need for change, there are few published examples of private hospitals that have successfully lowered their CS rates. Our hospital, serving private patients too, had a CS rate of 79% in 2001. Care was provided by fee-for-service visiting consultant obstetricians without uniform clinical protocols and little clinical governance. Consultants attributed high CS rate to case-mix and maternal demand and showed little inclination for change. We attempted to reduce this rate with the objective of improving the quality of our care and demonstrating that CS could be safely lowered in the private urban Indian healthcare setting.

**Methods:**

We hired full-time salaried consultants and began regular audit of CS cases. When this proved inadequate, we joined an improvement collaborative in 2011 and dedicated resources for quality improvement. We adopted practice guidelines, monitored outcomes by consultant, improved labour ward support, strengthened antenatal preparation, and moved to group practice among consultants.

**Results:**

Guidelines ensured admissions in active labour and reduced CS (2011 to 2016) for foetal heart rate abnormalities (23 to 5%; *p* < 0.001) and delayed progress (19 to 6%; *p* < 0.001) in low-risk first-birth women. Antenatal preparation increased trial of labour, even among women with prior CS (28 to 79%; *p* < 0.001). Group practice reduced time pressure and stress, with a decline in CS (52 to 18%; *p* < 0.001) and low-risk first-birth CS (48 to 12%; *p* < 0.001). Similar CS rates were maintained in 2017 and 2018. Measures of perinatal harm including post-partum haemorrhage, 3rd-4th degree tears, shoulder dystocia, and Apgar < 7 at 5 min were within acceptable ranges (13, 3, 2% and 3 per thousand respectively in 2016–18,).

**Conclusions:**

It is feasible to substantially reduce CS rate in private healthcare setting of a middle-income country like India. Ideas such as moving to full-time attachment of consultants, joining a collaborative, improving labour ward support, providing resources for data collection, and perseverance could be adopted by other hospitals in their own journey of moving towards a medically justifiable CS rate.

## Background

There is a global epidemic of caesarean sections (CS), which have increased from 12% in 2000 to about 21% in 2015 [1]. While many countries have a CS rate indicative of reduced access to this lifesaving procedure, the majority have a rate above the 10–15% range that is considered to be medically justifiable [2]. Even within countries there is often much variability in the CS rate based upon income level and place of delivery [3]. High CS rates are of concern because they expose the mother and child to short-term and long-term health risks [4] and impose a financial burden on families and health systems [5].

In line with global trends, the CS rate in India has increased from 8.5% in 2005–06 to 17.2% in 2015–16 [6]. In the same period the CS rate in private (including non-profit) facilities, which now account for more than 1 in 4 deliveries, has increased from 27.7 to 40.9% [6]. In some states the CS rate in private facilities is above 70% [7]. While the government has laid down standards for antenatal care such as minimum of four antenatal visits, iron supplementation, haemoglobin and urine testing, and tetanus toxoid injection, private urban facilities typically have more antenatal visits, multiple routine ultrasounds and limited emphasis on antenatal education [8, 9]. Overmedicalisation of birth as most visibly evident by the high CS rate is common.

Internationally, obstetricians’ fear of litigation, convenience, and declining skills for instrumental deliveries; preference for CS by higher-income group women; fee-for-service remuneration model in private healthcare; and lack of support from midwives had been identified as important factors for the rising CS rate [10]. Attempts to lower CS rate by issuing guidelines, providing uniform remuneration for both CS and vaginal deliveries, active management of labour, audit, mandatory second opinions, and expanding role of midwives had shown mixed results [10]. By the 1990s some authors had begun to raise the alarm around the issue of increasing CS in India but found few examples of hospitals that had tried similar interventions or successfully lowered their CS rate [10]. There seemed to be a widespread belief among Indian obstetricians that high rates were inevitable in private healthcare and there were no attempts by professional bodies to address this issue [10].

Our hospital in Delhi was also caught up in the caesarean epidemic with a CS rate of 79% for its 421 deliveries in 2001. We embarked on a journey to reduce this rate with the objective of improving the quality of our care and attempting to demonstrate that CS could be safely lowered in the private urban Indian healthcare setting.

## Methods

### Local setting

All deliveries in our 70-bed non-profit hospital, serving private patients, were conducted by fee-for-service consultant obstetricians assisted by junior obstetricians (who had recently obtained a post-graduate qualification) and nurses. The hospital had a 24-h functioning operating theatre and on-site presence of a paediatrician. All the consultant obstetricians were in solo practice and typically visited multiple hospitals. Women belonged to the upper socio-economic strata and paid for services either out-of-pocket or through private insurance. The hospital had no written clinical protocols and there was no practice of audit. Obstetricians seemed unaware of their CS rate and attributed the high rates to case-mix and maternal demand. Despite the high CS rate, women seemed satisfied with care and obstetricians showed little inclination for change. The hospital depended on consultants to bring in cases and could not insist upon a commitment for reducing CS.

A trigger for action came in the form of a formal complaint in 2001 by a woman who felt pressurised into having a prelabour caesarean for a medically unjustifiable indication.

### Approach

In 2002, the chief executive hired two consultant obstetricians, who had recently completed their training, with the explicit understanding that they would work together as a unit, especially with the intent of lowering the CS rate (Table 1). They were hired full-time, on a fixed salary, to enable them to focus their attention on developing an evidence-based maternity program without financial pressures. Lacking a clear understanding of what might be a medically justifiable CS rate for our hospital we picked a figure of 25% as a goal.

**Table 1:** Sequence of initiatives introduced for reducing caesarean section (CS) rate

Year	Initiatives
**2002**	• Created unit of full-time salaried consultant obstetricians • Began monthly audit of CS cases and review of medical literature • Started antenatal classes
**2003–08**	• Continued audit but frequency declined after 2005 • Sent consultants to observe care in other maternity and midwifery units • Invited obstetric expert for providing guidance on care practices
**2011**	• Joined improvement collaborative
**2012**	• Began monthly presentation of data within the unit on CS and other perinatal measures with focus on low-risk first birth mothers (Robson’s groups 1 and 2) and with consultant name unblinded • Recruited more experienced junior obstetricians for labour ward care • Reached consensus on management of abnormal fetal heart rate (FHR) as per standard guidelines and started regular FHR tracing review meetings
**2013**	• Drafted clinical guidelines for management of labour (admission, induction, augmentation, oxytocin administration, and others) • Revised labour documentation to enable comprehensive data capture • Trained and recruited additional nurses to enable of 1:1 support for all first-birth mothers in active labour
**2014**	• Drafted clinical guidelines for antenatal care • Strengthened antenatal preparation with additional childbirth counsellors and involvement of birth partners • Increased empowerment of junior obstetricians in the labour ward through use of structured communication with consultant obstetricians
**2015**	• Began group practice with select consultants enabling shared decision-making for challenging cases • Changed clinical leadership with “positive deviant” obstetrician becoming the lead
**2016–2018**	• Focused on promoting trial of labour after caesarean (TOLAC) • Consolidated group practice by involving all consultants

The consultants started monthly review of CS cases with a senior obstetrician, developed a few clinical protocols, and started antenatal classes. Simultaneously, we started regular review of contemporary international clinical guidelines, in the hope that it would change practice and lower CS rate.

In the first year, we were able to achieve a CS rate of 40%. But despite ongoing audit, visiting to other centres to observe care, attending training programmes, and inviting overseas experts to our hospital, we were unable to progress any further. With time, audit frequency decreased, and consultants began to practice individually rather than as a unit. In 2011, nine years after starting our initiative, we remained at a CS rate of above 50%.

### Relevant changes

Towards the middle of 2011, the chief executive decided to join an improvement collaborative as a way of refocusing on the goal of lowering CS rate and obtaining expert guidance [11]. This collaborative included several hospitals from the United States, which received monthly coaching for moving towards evidence-based maternity care and shared their progress. The collaborative used a set of interventions that had proven effective in lowering CS [12].

A team comprising the chief executive, 4 full-time salaried obstetricians, including one clinical director, and a manager with training in quality improvement was formed for this initiative.

The collaborative required detailed audit of our care protocols and clinical outcomes to identify gaps and select priority areas for improvement. The collaborative focused us on low-risk first-birth women (Nulliparous Term Single Cephalic; Robson’s groups 1 and 2) as an area of maximal impact (three-fourths of 52% CS in 2011 were contributed by low-risk first-birth women). We inducted two quality officers to assist with data collection (file review and clinician interview) and analysis.

Initial analysis revealed the following deficiencies: no structured system of data collection and audit, limited collaboration among consultants, absence or poor adherence to clinical protocols, inadequate nursing support in the labour room in terms of headcount and skill, and no standard algorithms for managing indeterminate foetal heart rate (FHR) patterns or delayed progress of labour, and no standard definition of harm.

Further investigation revealed that the labour room clinical record lacked enough detail to capture all relevant information. The existing clinical protocols had been created many years before and needed to be reviewed. Nurses were rotated between the labour room and general ward resulting in sub-optimal experience with birthing. Junior obstetricians had a high turnover, as they were typically looking for opportunities for further training, and in absence of standardised care they merely followed consultant orders. Antenatal classes were offered infrequently and there weren’t enough antenatal counselors in the outpatient clinic.

The labour room clinical record was revised using Plan-Do-Study-Act cycles over a period of 4 weeks. In absence of Indian guidelines, clinical guidelines from the Royal College of Obstetricians and Gynaecologists (UK), National Institute of Health and Care Excellence (UK), and the American College of Obstetricians and Gynecologists were adapted for use. Consequently, we created standard protocols for admission to the labour ward, induction, oxytocin administration, management of FHR abnormalities and other situations. All obstetricians took a formal course in electronic foetal monitoring and began regular review of FHR tracings in unit meetings.

Practice of rotating labour room nurses to the ward was stopped, additional nurses recruited, and a flexible roster introduced to ensure skilled 1:1 support for all first-time mothers in labour. Consultants developed a weekly calendar for nurse training. Junior obstetricians leaving the unit were replaced by those who had completed their training in teaching institutions and were thus more experienced and likely to have a longer tenure in the organisation.

Participation in the collaborative required monthly reporting of clinical outcomes and compliance to care practices. These measures included non-medically indicated delivery rate prior to 39 weeks, low-risk first-birth caesarean rate, transfer of babies to higher level of care, and compliance with care-bundles for induction and augmentation. Figures were shared within our unit with consultant name unblinded.

Weekly meetings involving consultants, junior obstetricians, select nurses and the quality manager and officers helped identify priority areas, reach consensus on change ideas, and review impact of changes. Every month we presented our progress to different hospital teams of the collaborative and had one-on-one sessions with the collaborative faculty. Several members of the improvement team and clinical staff attended in-person meetings of the collaborative during our 2 years of enrolment.

## Results

Participation in the collaborative led to transparency and understanding of our care processes and outcomes, and how these compared with best practices. Regular reporting and sharing outcomes by consultant name (within the unit) ensured sustained focus on the CS rate.

Adoption of clinical protocols, additional recruitment and training of nurses, and replacement of junior obstetricians by those with more experience reduced anxiety around management of labour. Use of structured communication by junior obstetricians promoted greater participation in clinical decision-making [13].

Compliance to guidelines for admission and oxytocin administration rose to near 100% soon after joining the collaborative. However, there was substantial variation in CS rate between consultants who continued to practice individually.

The CS rate decline in the initial 2 years (2012–13) was largely due to reduction achieved by one consultant, who attributed this improvement to antenatal sensitisation for a normal delivery, avoiding excessive weight gain, exercising to build stamina, and spending long hours with women during labour to compensate for weak nursing and junior obstetrician support. Two consultants, including the clinical director, left the unit to pursue other opportunities. The last of the original four consultants showed progress in reducing her caesarean rate 4 years (2015) after the start of the collaborative. The consultant leading the reduction in CS rate assumed leadership of the unit in 2015.

Three new consultants joining the unit in 2015–16 quickly adopted clinical protocols and commitment to promoting normal deliveries. They began group practice with shared decision-making for difficult cases, supporting each other for clinical responsibilities, and covering nights by rotation. With reduced consultant anxiety and time pressure, they became more confident about offering trial of labour after caesarean and the vaginal birth after caesarean rate improved. By the end of 2016, all consultants in the unit became part of group practice.

**Fig. 1:**
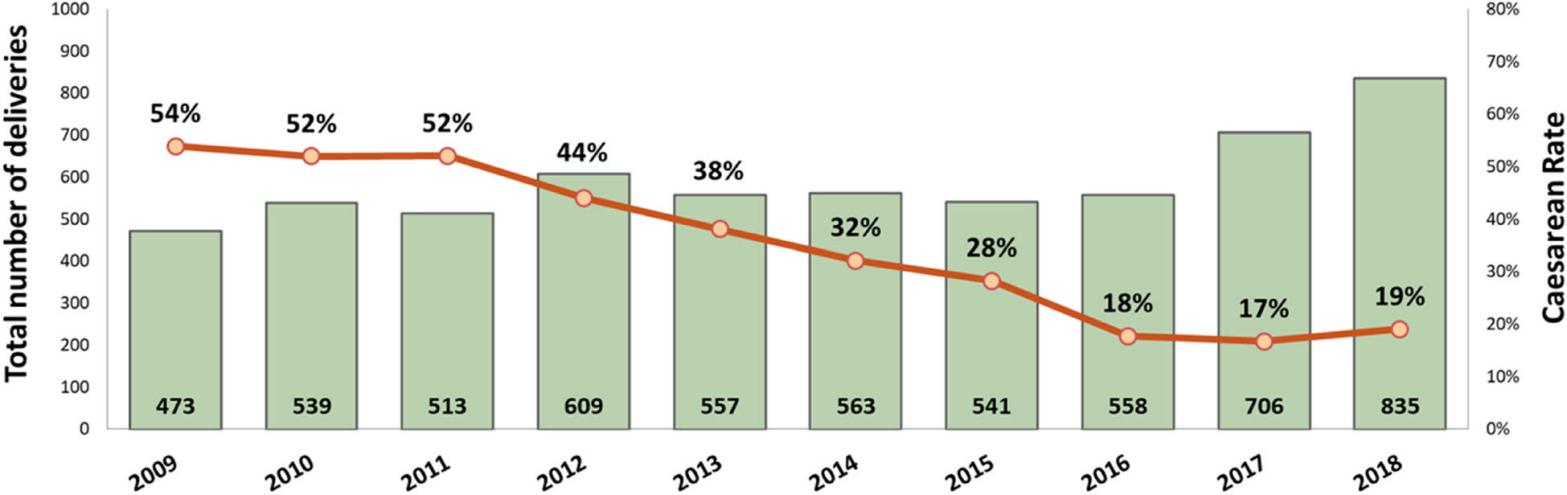
Annual caesarean section rate and delivery numbers from 2011 to 2018

**Fig. 2:**
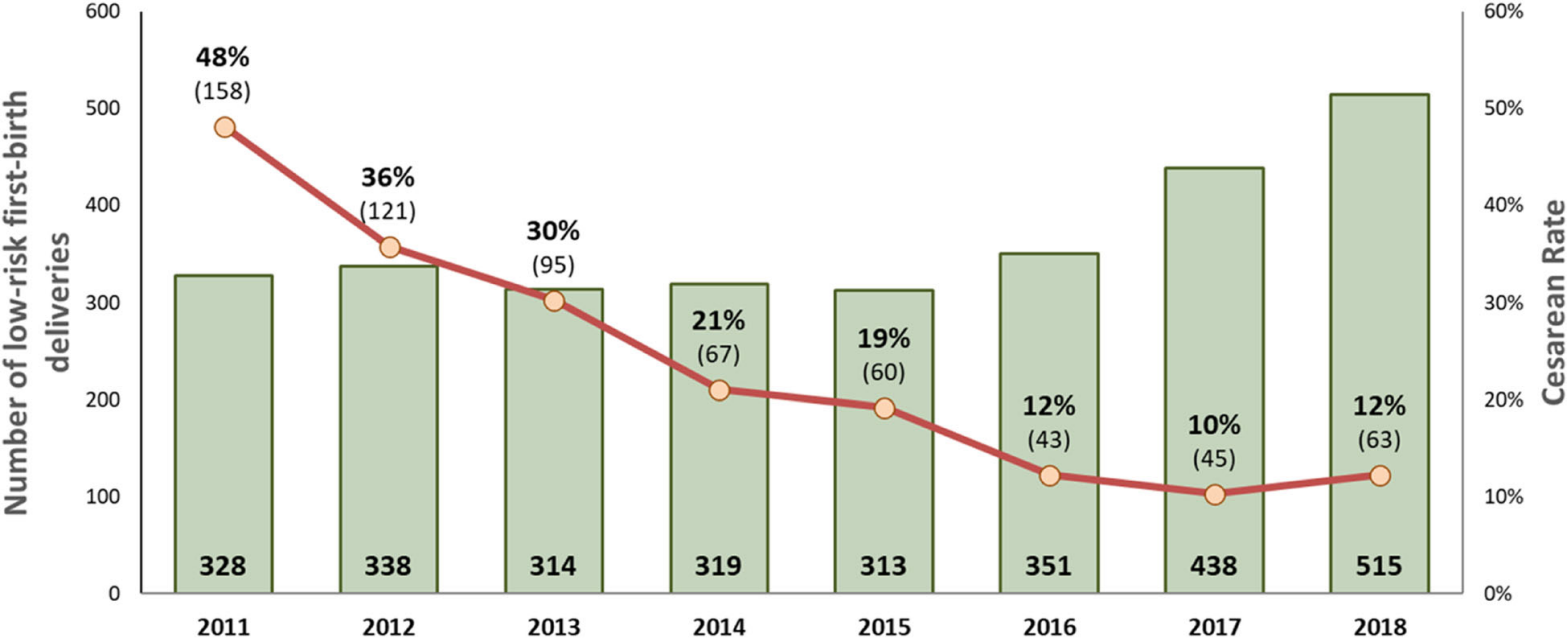
Low-risk first-birth (Robson’s Groups 1 and 2) CS rate

**Fig. 3:**
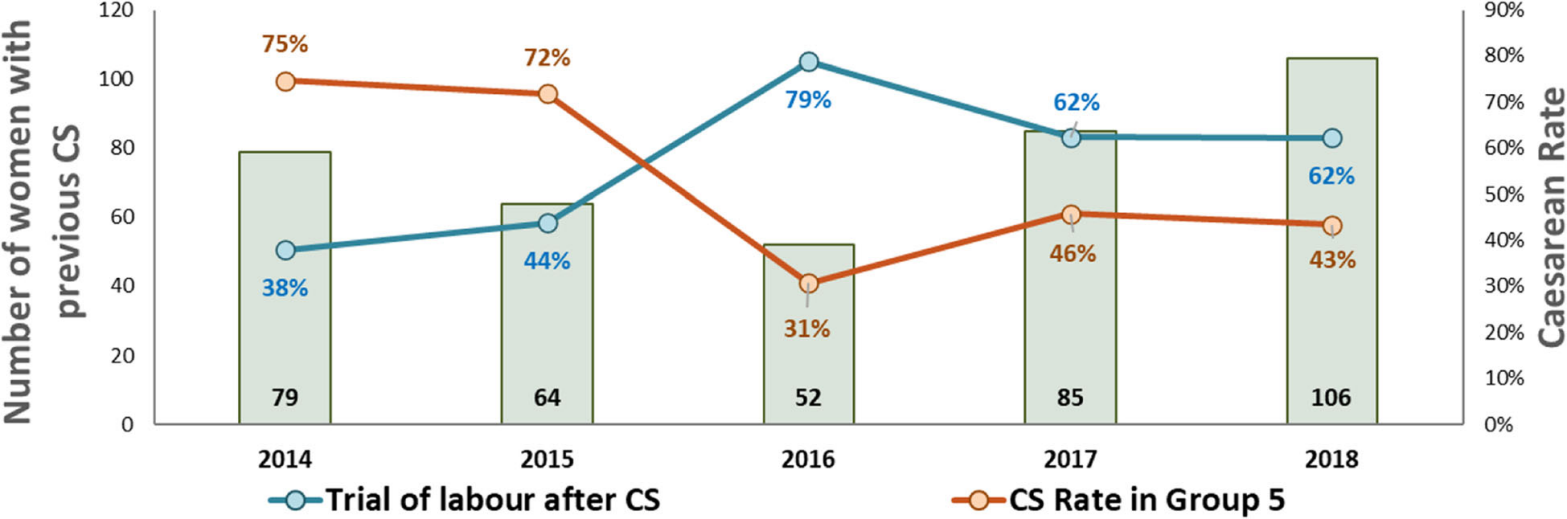
Trial of labour after caesarean and CS rate in women with previous caesarean (Robson’s Group 5)

**Fig. 4:**
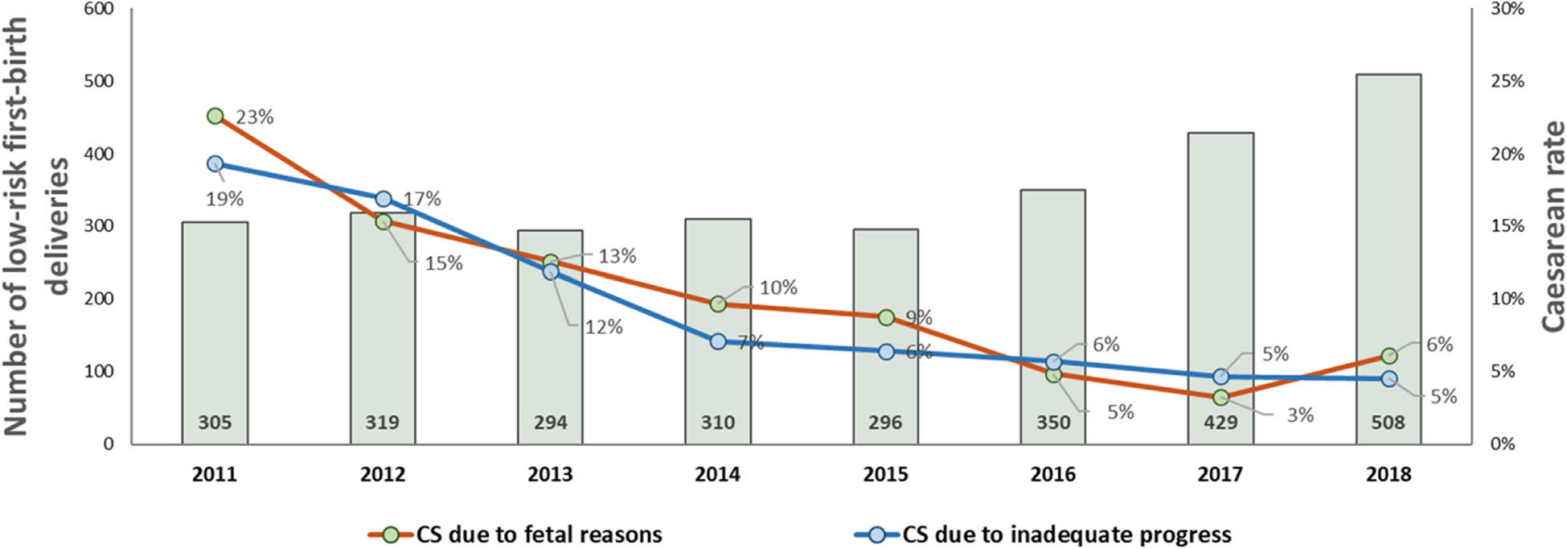
CS in low-risk first-birth women due to fetal reasons and inadequate progress

**Fig. 5:**
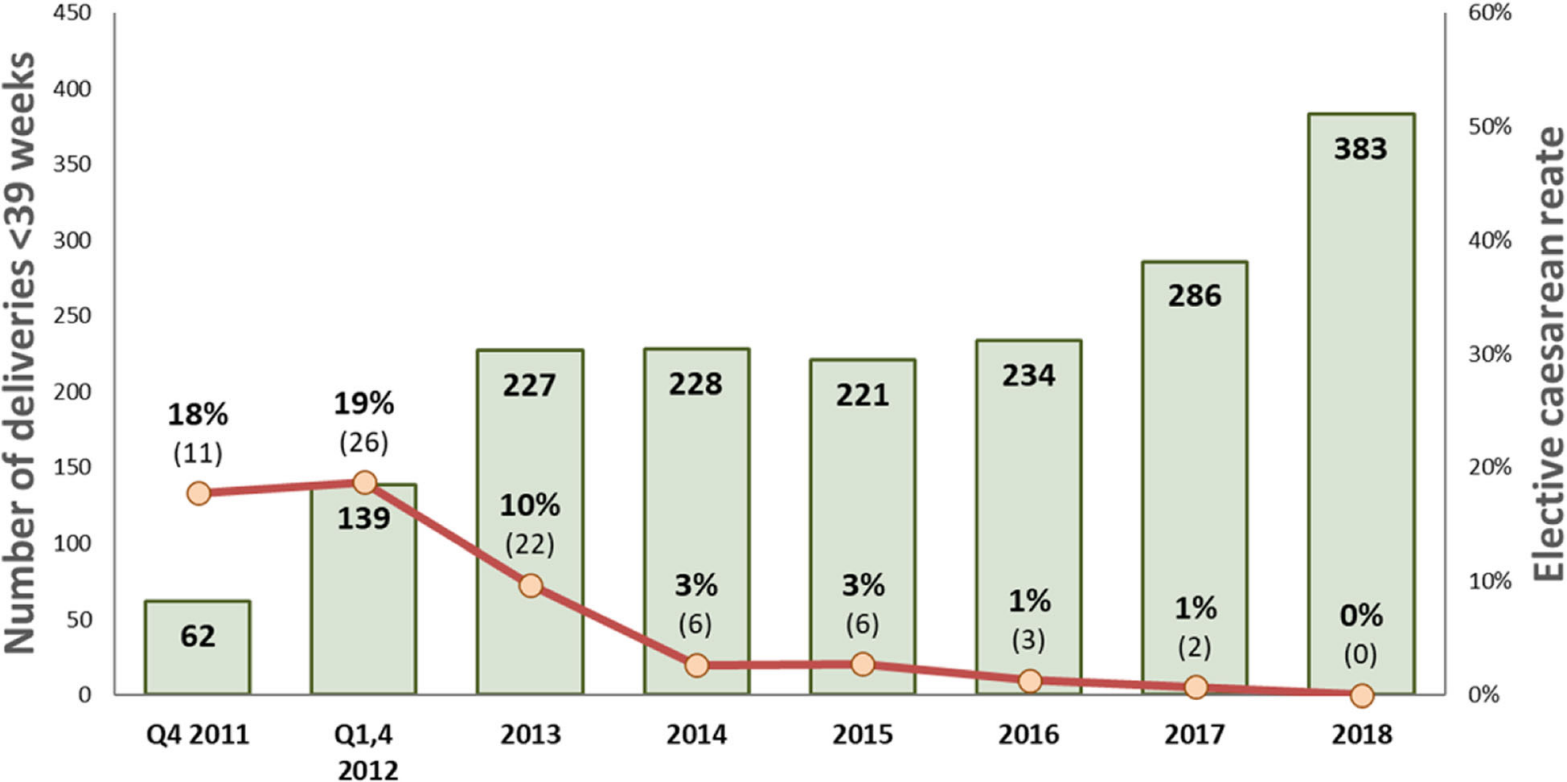
Elective delivery rate prior to 39 weeks

Between 2011 and 2016 (Fig. 1), the CS rate declined from 52 to 18%, (*p* < 0.001). The greatest declines were for low-risk first-birth women (Robson’s groups 1 and 2; 48 to 12%, *p* < 0.001; Fig. 2) and for term-single-cephalic women with previous caesareans (Robson’s group 5; 90 to 31%, *p* < 0.001; Fig. 3). Emergency caesareans for suspected FHR abnormalities (23 to 5% of low-risk first-birth women, *p* < 0.001) and delayed progress of labour (19 to 6%, *p* < 0.001; Fig. 4) also declined. Women undergoing a trial of labour after caesarean increased (28 to 79%, *p* < 0.001; Fig. 4). Elective deliveries prior to 39 weeks were almost eliminated (18% in 2011–12 to 1% in 2016, *p* < 0.001; Fig. 5). The lower CS rates were maintained in 2017 and 2018. Measures of perinatal harm including post-partum haemorrhage, 3rd-4th degree tears, shoulder dystocia, and Apgar < 7 at 5 min were within acceptable ranges (13, 3, 2% and 3 per thousand in 2016–18, respectively). Women’s satisfaction with care was high; > 95% gave a rating of ≥4 on a 5-point scale. In contrast, the CS rate of fee-for-service visiting consultants increased (*p* = 0.02) marginally from 2011 (206/321; 64%) to 2016–18 (297/412;72%).

## Discussion

To the best of our knowledge, this is a unique report of an urban hospital serving private patients in India engaging in systematic efforts for successfully lowering its caesarean rate. In comparison to an average of 54% in Delhi’s private hospitals, we recorded CS rates of 17–19% during 2016–2018 [9]. Moving from a fee-for-service attachment of consultants to full-time employment changed the customer-hospital dynamic that often characterises the relationship between consultants and hospitals in private healthcare and acts as a barrier for clinical governance. Emergence of a positive deviant consultant who was able to demonstrate that lower rates were possible with “our patients, in our setting” proved critical. Other important contributors for this success included joining an improvement collaborative, hiring new medical and nursing staff, providing quality improvement support, continual close involvement of chief executive, and perseverance over 15 years.

We were able to succeed because we comprehensively addressed barriers identified by private obstetricians for lowering CS rates – maternal and family attitude, time pressure created by solo practice with attachment to multiple hospitals, inadequate labour ward support, ambiguity of clinical management due to absence of Indian practice guidelines, and lack of accountability for high CS due to absence of measurement and reporting [14]. Critically, we benefited from emergence of clinical leadership that believed in promoting normal birth and from sustained organisational commitment to this philosophy, thus shaping obstetricians’ views, which are important for determining CS rate [15]. Full-time attachment at one hospital and group practice helped counter the use of CS for obstetrician convenience, another factor increasing CS [16]. Sharing outcomes by name of consultant, as recommended by other quality improvement collaboratives, created accountability and was likely contributor to lowering CS [17]. Despite organisational support, fear of criticism and litigation for not conducting a CS in the event of an adverse outcome remained a major concern among our consultants.

An important limitation is that our experience was not based on randomised controlled design, which compromises inferences on causality. However, the visiting fee-for-service consultant comparison provides a control arm similar to a quasi-randomised study. Visiting consultants did not participate in this initiative; the marginal increase in their CS rate during the same period suggests a causal role of the intervention package. Further, with multiple overlapping interventions in an evolving context, we cannot comment on the relative contribution of various aspects.

The generalisability of our experience may be limited to relatively well-resourced private hospitals in India or other countries with similar health systems. Smaller hospitals may not be able to hire full-time consultants or increase staffing in the labour ward. However, policy changes such as requiring public disclosure of CS rate, as is now being initiated in India, might create the accountability needed to spur action [18]. Given the multiple barriers discussed above, accountability alone is unlikely to be sufficient without other enabling conditions. Introducing midwifery as an independent profession and strengthening midwifery skills of nurses who support obstetrician-led care may prove to be one of the most effective interventions for reducing CS in the long-term [19].

## Conclusion

With determined systematic efforts, it is feasible to substantially reduce CS rate in private healthcare setting of a middle-income country like India where oversight of medical practice tends to be weak. Interventions such as moving to full-time attachment of consultants, joining a collaborative, improving labour ward support, providing resources for data collection, and committing to change over a long period could be adopted by other hospitals in their own journey of moving towards a medically justifiable CS rate.

## Data Availability

The datasets used and/or analysed during the current study are available from the corresponding author on reasonable request.

## Abbreviations

CS: Caesarean Section
FHR: Foetal Heart Rate
TOLAC: Trial of Labour After Caesarean
